# Hierarchical composite outcomes in acute ischaemic stroke with large infarct: a win ratio analysis of the TENSION trial

**DOI:** 10.1093/esj/aakag063

**Published:** 2026-06-18

**Authors:** Olga Ciobanu-Caraus, Philip Heesen, Michael D Hill, Susanne Bonekamp, Jens Fiehler, Anne Hege Aamodt, Blanca Fuentes, Elke R Gizewski, Antonin Krajina, Laurent Pierot, Claus Z Simonsen, Kamil Zeleňák, Rolf A Blauenfeldt, Bastian Cheng, Angélique Denis, Hannes Deutschmann, Franziska Dorn, Fabian Flottmann, Susanne Gellißen, Johannes C Gerber, Mayank Goyal, Jozef Haring, Christian Herweh, Silke Hopf-Jensen, Vi Tuan Hua, Märit Jensen, Andreas Kastrup, Christiane Fee Keil, Andrej Klepanec, Egon Kurča, Ronni Mikkelsen, Stefan Müller-Hülsbeck, Nico Münnich, Paolo Pagano, Panagiotis Papanagiotou, Gabor C Petzold, Mirko Pham, Volker Puetz, Jan Raupach, Gernot Reimann, Peter Arthur Ringleb, Maximilian Schell, Eckhard Schlemm, Silvia Schönenberger, Bjørn Tennøe, Christian Ulfert, Kateřina Vališ, Eva Vítková, Dominik F Vollherbst, Wolfgang Wick, Götz Thomalla, Markus Möhlenbruch, Martin Bendszus, TENSION Investigators, Anne Hege Aamodt, Olaf Adamczewski, Kirill Alektoror

**Affiliations:** Interventional Neuroradiology, Department of Neuroradiology, Heidelberg University Hospital, Heidelberg, Germany; Interventional Neuroradiology, Department of Neuroradiology, Heidelberg University Hospital, Heidelberg, Germany; Department of Biostatistics at the Epidemiology, Biostatistics and Prevention Institute, University of Zurich, Zurich, Switzerland; Department of Clinical Neurosciences, Hotchkiss Brain Institute, Health Science Centre, University of Calgary & Foothills Medical Centre, Calgary, Alberta, Canada; Interventional Neuroradiology, Department of Neuroradiology, Heidelberg University Hospital, Heidelberg, Germany; Klinik und Poliklinik für Neuroradiologische Diagnostik und Intervention, Universitätsklinikum Hamburg-Eppendorf, Hamburg, Germany; Eppdata GmbH, Hamburg, Germany; Department of Neurology, Oslo University Hospital, Oslo, Norway; Department of Neurology and Stroke Centre, Hospital La Paz Institute for Health Research-IdiPAZ (La Paz University Hospital-Universidad Autónoma de Madrid), Madrid, Spain; Department of Radiology, Medical University Innsbruck, Innsbruck, Austria; Department of Radiology, Faculty of Medicine in Hradec Králové, Charles University, Hradec Králové, Czech Republic; Department of Neuroradiology, Hôpital Maison-Blanche, Université Reims-Champagne-Ardenne, Reims, France; Department of Neurology, Aarhus University Hospital, Aarhus, Denmark; Clinic of Radiology, Jessenius Faculty of Medicine, Comenius University, Martin, Slovakia; Department of Neurology, Aarhus University Hospital, Aarhus, Denmark; Klinik und Poliklinik für Neurologie, Universitätsklinikum Hamburg-Eppendorf, Hamburg, Germany; Service deBiostatistique, Hospices Civils de Lyon, Lyon, France; Laboratoire de Biométrie et Biologie Évolutive, Université de Lyon, Villeurbanne, France; Division of Neuroradiology, Vascular and Interventional Radiology, Department of Radiology, Medical University Graz, Graz, Austria; Klinik für Diagnostische und Interventionelle Neuroradiologie, Universitätsklinikum Bonn, Bonn, Germany; Klinik und Poliklinik für Neuroradiologische Diagnostik und Intervention, Universitätsklinikum Hamburg-Eppendorf, Hamburg, Germany; Klinik und Poliklinik für Neuroradiologische Diagnostik und Intervention, Universitätsklinikum Hamburg-Eppendorf, Hamburg, Germany; Institute of Neuroradiology, Universitätsklinikum Carl Gustav Carus an der Technischen Universität Dresden, Dresden, Germany; Dresden Neurovascular Center, Universitätsklinikum Carl Gustav Carus an der Technischen Universität Dresden, Dresden, Germany; Department of Clinical Neurosciences, Hotchkiss Brain Institute, Health Science Centre, University of Calgary & Foothills Medical Centre, Calgary, Alberta, Canada; Department of Neurology, Faculty Hospital Trnava, Trnava, Slovakia; Interventional Neuroradiology, Department of Neuroradiology, Heidelberg University Hospital, Heidelberg, Germany; Institut für Diagnostische und Interventionelle Radiologie und Neuroradiologie, DIAKO Krankenhaus gGmbH, Flensburg, Germany; Department of Neuroradiology, Hôpital Maison-Blanche, Université Reims-Champagne-Ardenne, Reims, France; Klinik und Poliklinik für Neurologie, Universitätsklinikum Hamburg-Eppendorf, Hamburg, Germany; Klinik für Neurologie, Klinikum Bremen Mitte, Bremen, Germany; Institut für Neuroradiologie, Universitätsklinikum Frankfurt, Frankfurt am Main, Germany; Department of Radiology, Faculty Hospital Trnava, Trnava, Slovakia; Clinic of Neurology, Jessenius Faculty of Medicine, Comenius University, Martin, Slovakia; Department of Neuroradiology, Aarhus University Hospital, Aarhus, Denmark; Institut für Diagnostische und Interventionelle Radiologie und Neuroradiologie, DIAKO Krankenhaus gGmbH, Flensburg, Germany; Klinikum Dortmund gGmbH, Klinikum der Universität Witten/Herdecke, Dortmund, Germany; Department of Neuroradiology, Hôpital Maison-Blanche, Université Reims-Champagne-Ardenne, Reims, France; Klinik für Diagnostische und Interventionelle Neuroradiologie, Klinikum Bremen Mitte, Bremen, Germany; Department of Radiology, Aretaieion University Hospital, National and Kapodistrian University of Athens, Athens, Greece; Vascular Neurology Research Group, German Center for Neurodegenerative Diseases (DZNE), Bonn, Germany; Division of Vascular Neurology, Department of Neurology, University Hospital Bonn, Bonn, Germany; Institut für Diagnostische und Interventionelle Neuroradiologie, Universitätsklinikum Würzburg, Würzburg, Germany; Dresden Neurovascular Center, Universitätsklinikum Carl Gustav Carus an der Technischen Universität Dresden, Dresden, Germany; Department of Neurology, Universitätsklinikum Carl Gustav Carus an der Technischen Universität Dresden, Dresden, Germany; Department of Radiology, Faculty of Medicine in Hradec Králové, Charles University, Hradec Králové, Czech Republic; Klinikum Dortmund gGmbH, Klinikum der Universität Witten/Herdecke, Dortmund, Germany; Neurologie, Universitätsklinikum Heidelberg, Heidelberg, Germany; Klinik und Poliklinik für Neurologie, Universitätsklinikum Hamburg-Eppendorf, Hamburg, Germany; Klinik und Poliklinik für Neurologie, Universitätsklinikum Hamburg-Eppendorf, Hamburg, Germany; Neurologie, Universitätsklinikum Heidelberg, Heidelberg, Germany; Department of Neuroradiology, Oslo University Hospital, Oslo, Norway; Interventional Neuroradiology, Department of Neuroradiology, Heidelberg University Hospital, Heidelberg, Germany; Department of Medical Imaging, St. Anne’s University Hospital Brno and Faculty of Medicine, Masaryk University, Brno, Czech Republic; Department of Neurology, Faculty of Medicine in Hradec Králové, Charles University, Hradec Králové, Czech Republic; Interventional Neuroradiology, Department of Neuroradiology, Heidelberg University Hospital, Heidelberg, Germany; Neurologie, Universitätsklinikum Heidelberg, Heidelberg, Germany; Klinik und Poliklinik für Neurologie, Universitätsklinikum Hamburg-Eppendorf, Hamburg, Germany; Interventional Neuroradiology, Department of Neuroradiology, Heidelberg University Hospital, Heidelberg, Germany; Interventional Neuroradiology, Department of Neuroradiology, Heidelberg University Hospital, Heidelberg, Germany

**Keywords:** acute ischaemic stroke, large infarct, win ratio

## Abstract

**Introduction:**

Traditional endpoints in acute ischaemic stroke trials, most commonly the mRS, incompletely capture the full spectrum of patient outcomes. The win ratio (WR) is a hierarchically structured composite outcome measure that prioritises endpoints according to clinical importance. In this study, we applied win statistics to investigate which components drive the overall treatment effect.

**Patients and methods:**

Of 253 patients in the TENSION (Efficacy and safety of ThrombEctomy iN Stroke with extended leSION and extended time window) trial, 125 were randomised to EVT + BMT and 128 to BMT alone. Endpoints were ranked hierarchically: (1) time to death, (2) mRS at 12 months, (3) occurrence of any serious adverse event and (4) EuroQOL 5-dimension scores at 12 months. Outcomes were analysed using win statistics.

**Results:**

The overall WR was 1.61 (95% CI, 1.19–2.18; *P* = .002), indicating a relative frequency of wins for EVT + BMT of approximately 60% among untied pairwise comparisons. The win difference was 22.8% (95% CI, 8.3%–36.3%; *P* = .002), of which 15.7% were attributable to survival. Win ratios were consistent across prespecified subgroups, including both sexes, age ≤ 80 years, baseline mRS 0–1, conscious patients, ASPECTS 5 and M1 occlusions.

**Discussion:**

The WR provides an informative complement to conventional mRS-based analyses in acute ischaemic stroke trials. Our hierarchical composite outcome analysis demonstrated how ordering of hierarchical components affects the resulting estimates, with the predominant treatment effect in TENSION being driven by improved survival.

**Conclusion:**

These findings support the complementary use of hierarchical composite outcomes in future neurointerventional trials.

## Graphical Abstract

**Graphical Abstract:**
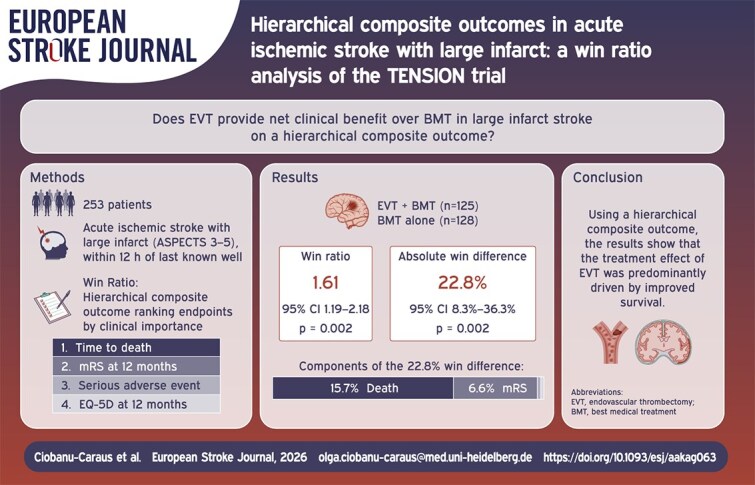

## Introduction

The TENSION (Efficacy and safety of ThrombEctomy iN Stroke with extended leSION and extended time window) trial^1^ compared EVT plus best medical treatment (BMT) vs BMT alone in patients with acute ischaemic stroke and large infarcts and demonstrated that EVT significantly improved functional outcomes, with median mRS at 90 days as the primary outcome.

In trials on acute ischaemic stroke—as in the TENSION trial—it is common to report ordinal outcomes such as the mRS as the primary endpoint.^1–3^ While simple and validated, the mRS lacks granularity to detect subtle yet clinically relevant changes in functional status. Early landmark thrombectomy trials dichotomised mRS outcomes, typically defining functional independence as mRS 0–2.^4^^,^^5^ However, dichotomisation compresses the full spectrum of functional outcomes, may under- or overestimate a treatment’s true effect and generally requires larger sample sizes to detect treatment effects.^6^ Given these limitations, an increasing number of experts have questioned whether mRS is an adequate primary endpoint for neurointerventional trials and advocated for the broader adoption of complementary outcome measures.^6^^,^^7^

Composite outcome measures offer an alternative approach by integrating multiple outcomes of varying clinical severity, including death, functional outcome, adverse events and quality of life.^8^ The win ratio (WR) approach, first proposed by Pocock et al.^9^ in 2012, represents a hierarchical method to analyse such composite outcomes by prioritising individual endpoints according to their clinical importance.^10^ This sequential comparison integrates outcomes of different types (eg, ordinal, continuous and time-to-event) into one single, hierarchically structured composite outcome measure which confers the overall “net” clinical benefit.^11^ While the WR has been increasingly adopted in cardiovascular medicine^12–14^ and has recently also been proposed for stroke research,^15^ its application in neurointerventional trials remains limited.

The aim of this study was to apply win statistics to the 12-month outcome data of the TENSION trial to investigate components that drive the overall treatment effect.

## Patients and methods

### Study population

This study used individual patient data from the TENSION trial,^1^ a multicentre, randomised, open-label, blinded-endpoint trial that compared EVT plus best medical treatment (BMT) vs BMT alone in patients with acute ischaemic stroke caused by anterior circulation LVO and large infarct core. Eligible patients were aged ≥ 18 years, had a baseline ASPECTS of 3–5 and were treated within 12 h from last known well.^1^ All participants or their legal representatives provided written informed consent. The original trial was approved by local ethics committees. The current analysis was approved by the TENSION Steering Committee and conducted in accordance with the Declaration of Helsinki.

### Statistical analysis

#### Demographic and clinical characteristics

Descriptive statistics were calculated for baseline demographic and clinical characteristics, including sex, age (≤70, 71–80 and > 80 years), NIHSS score at hospital admission, pre-stroke (baseline) mRS score, level of consciousness on arrival, baseline Alberta Stroke Program Early CT Score (ASPECTS), occlusion site (internal carotid artery [ICA], MCA M1 segment, MCA M2 segment or combined MCA + ACA), and thrombolysis (intravenous alteplase administered). Continuous variables are presented as median and IQR, categorical variables as counts and percentages.

#### Definition of hierarchical outcomes

Efficacy and safety outcomes were selected based on clinical importance and availability in the original dataset. The prespecified hierarchical ranking of outcomes, from highest to lowest priority, was prespecified as follows: (1) time to death, (2) median mRS at 12-month follow-up, (3) occurrence of any serious adverse event (SAE), as defined in the original protocol and (4) quality of life, measured by EuroQOL group 5-dimension (EQ-5D) score. The hierarchy of these components was determined by consensus among members of the TENSION Steering Committee. In the primary publication, safety endpoints were descriptively presented in the per-protocol population. For the present analysis, we calculated WR based on the intention-to-treat population. Therefore, safety endpoints were also derived from the intention-to-treat-population, and the descriptive values are presented accordingly.

#### Unstratified win ratio

Treatment effects of EVT + BMT compared with BMT alone were evaluated using the WR method based on the methods proposed by Peron et al.^16^ Each EVT + BMT patient was compared pairwise with every BMT patient, generating an all-pairs dataset (*n* = 16,000). Within each pair, outcomes were compared sequentially using the prespecified hierarchical decision tree, and each pair was assigned a “win,” “loss” or “tie.” A pair was classified as a “win” if a participant randomised to the EVT + BMT group experienced a better outcome, and as a “loss” if vice versa. A pair was tied if the pair had the same outcome. For time-to-event outcomes (survival), only deaths occurring within each pair’s shared follow-up period were considered. If both died, the patient with a longer survival was considered a “winner.” The WR was calculated as the number of wins in the EVT + BMT group divided by the number of losses, and the 95% CI was calculated using a non-parametric bootstrap (*n* = 10,000). The WR ranges between 0 (no winner pairs) and infinity (no loser pairs). A WR > 1 indicates a positive treatment effect in the EVT + BMT group. This can be interpreted as follows: for any randomly chosen pair of patients (EVT + BMT and BMT alone) that is not tied, the WR is the estimated odds that the patient treated with EVT + BMT will experience a better outcome. To estimate the absolute treatment effect, the win difference was calculated as (wins − losses)/(wins + losses + ties).^17^ It refers to the difference in the number of pairs that favour intervention rather than control among 100 randomly chosen patient pairs. This WR analysis followed the intention-to-treat principle.

#### Stratified win ratio

Separate WR analyses were performed within predefined strata. Subgroups included sex (male, female), age (≤70 years, 71–80 years, > 80 years), baseline mRS (mRS 0–1, mRS ≥ 2), level of consciousness on arrival (fully awake, somnolent/coma), baseline ASPECTS value (3, 4, 5), occlusion location (ICA, M1 segment) and thrombolysis (yes, no). Since no significant differences in baseline characteristics were observed between the treatment groups, no additional adjustments or matching procedures were performed.

#### Sensitivity analyses

To assess the implications of different permutations of the hierarchical ordering on the treatment effect, we conducted sensitivity analyses with modified hierarchical composite outcomes. First, we ranked functional outcome higher than death: (1) mRS at 12-month follow-up, (2) time to death, (3) occurrence of any SAE and (4) quality of life, measured by EQ-5D. Second, we additionally ranked quality of life higher than SAE: (1) mRS at 12-month follow-up, (2) time to death, (3) quality of life, measured by EQ-5D and (4) occurrence of any SAE.

#### Software

All analyses were performed in R Statistical Software (version 4.5.1, R Foundation for Statistical Computing, Vienna, Austria), using the *BuyseTest* package. Statistical significance was defined as a 2-sided *P*-value of < .05.

## Results

### Demographic characteristics and treatment outcomes

A total of 253 patients were randomised in the TENSION trial,^1^ all of whom were included in the WR analyses. Of these, 125 were randomised to EVT + BMT, and 128 to BMT alone. Baseline demographic and disease-specific characteristics stratified by EVT + BMT and BMT are detailed in Table 1. At 12 months, patients treated with EVT + BMT had a lower median (IQR) mRS score compared with those receiving BMT alone (5 [3–6] vs 6 [4–6]). Functional independence (mRS ≤ 2) was achieved in 22% of patients in the EVT + BMT group and 5% in the BMT group. Mortality was lower among patients treated with EVT + BMT (45%) than in those receiving BMT alone (56%). Similarly, serious adverse events occurred less frequently in the EVT + BMT group (54%) compared with the BMT group (71%) (Table 2).

**Table 1:** Baseline demographic and disease-specific characteristics of the study population stratified by EVT + BMT and BMT alone.

Characteristic	EVT + BMT *n* = 125	BMT only *n* = 128
**Female, *n* (%)**	56 (45)	67 (52)
**Age [years], *n* (%)**		
**≤ 70**	51 (41)	53 (41)
**71–80**	43 (34)	44 (34)
**> 80**	31 (25)	31 (24)
**NIHSS score at hospital arrival, median (IQR)**	19 (16–22)	18 (15–22)
**Pre-stroke mRS score, median (IQR)**	0 (0–1)	0 (0–1)
**Level of consciousness on hospital arrival, *n* (%)**		
**Fully awake**	76 (61)	79 (62)
**Somnolent/Coma**	47 (38)	47 (37)
**ASPECTS value, *n* (%)**		
**3**	36 (29)	48 (38)
**4**	45 (36)	39 (30)
**5**	44 (35)	41 (32)
**Occlusion site, *n* (%)**		
**ICA**	45 (33)	37 (29)
**MCA, M1 segment**	83 (66)	88 (69)
**MCA, M2 segment**	0 (0)	1 (1)
**MCA + ACA**	1 (1)	1 (1)
**Intravenous alteplase administered, *n* (%)**	49 (39)	44 (34)

Abbreviations: ACA, anterior cerebral artery; ASPECTS, Alberta Stroke Program Early CT Score; BMT, best medical treatment; ICA, internal carotid artery.

**Table 2:** Efficacy and safety outcomes stratified by EVT + BMT and BMT alone.

Outcome	EVT + BMT	BMT only
	*n* = 125	*n* = 128
**Primary outcome**		
** mRS at 12 months, median (IQR)**	5 (3–6)	6 (4–6)
**Secondary outcomes**		
** Independent functional outcome (mRS ≤ 2) at 12 months, *n* (%)**	27 (22)	7 (5)
** Death or dependency (mRS 4–6) at 12 months, *n* (%)**	84 (67)	98 (77)
** EQ-5D index score at 12 months**	0.7 (0.4–0.9)	0.4 (0.2–0.7)
**Safety outcomes**		
Overall survival (deaths, censored at 12 months)^*^, *n* (%)	58 (55)	70 (44)
**At least one serious adverse event, *n* (%)**	68 (54)	91 (71)

Abbreviations: BMT, best medical treatment; EQ-5D, EuroQOL 5-dimension; SAE, serious adverse event. ^*^Kaplan-Meier estimate.

### Unstratified win ratio analysis

**Figure 1:**
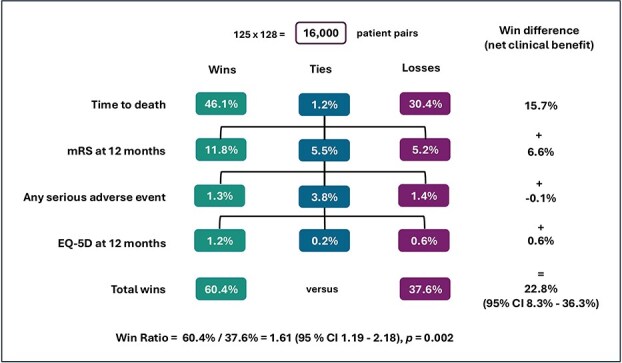
Hierarchical structure of the WR analysis. This schematic illustrates the hierarchical comparison process used in the WR analysis. Each patient treated with EVT + BMT is pairwise compared with every patient that received BMT alone. Outcomes are evaluated sequentially: (1) time to death, (2) mRS at 12 months, (3) any serious adverse event and (4) EQ-5D at 12 months. For each comparison, the pair is classified as an EVT + BMT “win,” “loss” or “tie” based on the first outcome in the hierarchy that differs between the 2 patients. The overall WR is calculated as the total number of EVT + BMT wins divided by total BMT wins, with corresponding 95% CI and *P*-value. This hierarchical approach summarises efficacy and safety across multiple clinically important endpoints into a single composite measure. Abbreviation: BMT = best medical treatment; EQ-5D = EuroQOL 5-dimension.

In the unstratified WR analysis, EVT + BMT won in 60.4% (9666/16,000) of paired comparisons and BMT alone in 37.6% (6018/16,000), with 2.0% (315/16,000) of comparisons tied. Overall, the WR was 1.61 (95% CI, 1.19–2.18; *P* = .002). The win difference was 22.8% (95% CI, 8.3%–36.3%; *P* = .002), of which 15.7% were attributable to a benefit in survival. The distribution of wins and ties for each paired comparison for the individual components of the hierarchical composite outcome is illustrated in Figure 1.

### Stratified win ratio analysis

**Figure 2:**
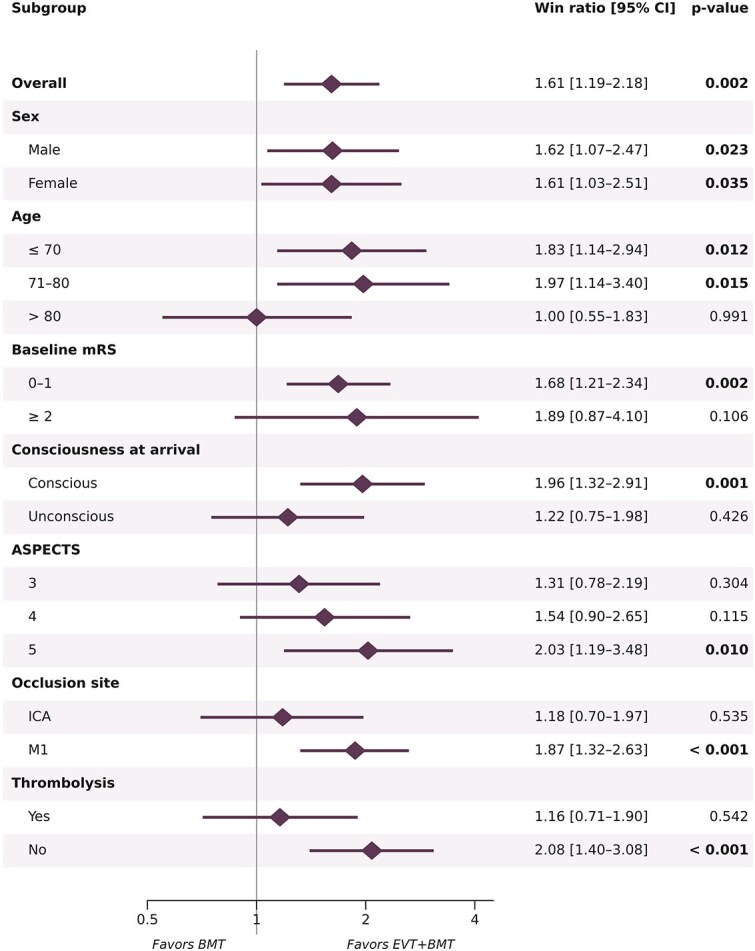
Forest plot showing the results of the unstratified and stratified WR analysis for EVT + BMT vs BMT alone. A WR > 1 indicates a positive treatment effect for EVT + BMT compared to BMT alone. The vertical dashed line represents the line of no difference (WR = 1). Abbreviations: BMT = best medical treatment; WR = win ratio.

The results of the stratified WR analysis are displayed in Figure 2. Stratified by sex, the WR was 1.62 (95% CI, 1.07–2.47; *P* = .023) for male patients and 1.61 (95% CI, 1.03–2.51; *P* = .035) for female patients. Across age groups, the WR was 1.83 (95% CI, 1.14–2.94; *P* = .012) for patients aged ≤ 70 years, 1.97 (95% CI, 1.14–3.40; *P* = .015) for those aged 71–80 years and 1.00 (95% CI, 0.55–1.83; *P* = .991) for patients aged > 80 years. When stratified by baseline mRS, the WR was 1.68 (95% CI, 1.21–2.34; *P* = .002) for patients with a baseline mRS of 0–1 and 1.89 (95% CI, 0.87–4.10; *P* = .106) for those with a baseline mRS ≥ 2. For patients who were fully conscious at arrival, the WR was 1.96 (95% CI, 1.32–2.91; *P* = .001), whereas for those presenting unconscious, the WR was 1.22 (95% CI, 0.75–1.98; *P* = .426). According to baseline ASPECTS, the WR was 1.31 (95% CI, 0.78–2.19; *P* = .304) for ASPECTS 3, 1.54 (95% CI, 0.90–2.65; *P* = .115) for ASPECTS 4 and 2.03 (95% CI, 1.19–3.48; *P* = .010) for ASPECTS 5. By occlusion site, the WR was 1.18 (95% CI, 0.70–1.97; *P* = .535) for ICA occlusions and 1.87 (95% CI, 1.32–2.63; *P* < .001) for M1 occlusions. Stratified by thrombolysis status, the WR was 1.16 (95% CI, 0.71–1.90; *P* = .542) among patients receiving IV thrombolysis and 2.08 (95% CI, 1.40–3.08; *P* < .001) among those who did not.

### Sensitivity analysis

Using modified hierarchical outcomes, the WRs in the overall group were 1.63 (95% CI, 1.21–2.21, *P* = .001) and 1.65 (95% CI, 1.22–2.22, *P* = .001), respectively (Figures S1 and S2). The results of the stratified WR analyses with the modified hierarchical outcomes are presented in Tables S1 and S2.

## Discussion

Traditionally, thrombectomy trials^2^^,^^3^ have reported dichotomised outcomes such as functional independence. However, dichotomised mRS scores fail to capture the full range of functional outcomes and may inherently under- or overestimate a treatment’s true effect. Modern landmark thrombectomy trials^18^^,^^19^ calculated the changes across the distribution of mRS scores—so-called “shift analysis.” However, the ability to detect treatment effects depends on the baseline functional status of randomised patients, which diminishes the probability of establishing a treatment benefit in patients with mild deficits. In addition, these broad scales are not able to capture domain-specific subtle improvements—eg, a musician or proceduralist regaining fine-motor skills—which may lead to a substantial increase in quality of life but not sufficient to shift the overall mRS grade.^6^ Moreover, conventional reporting cannot adequately capture various outcomes of different severity and in particular obscures the competing risk of death. For example, death can often occur after a non-fatal event (eg, aspiration pneumonia) rather than due to the ischaemic brain damage, which means that death may be underrepresented in primary outcomes. When ordinal scale scores include death (eg, mRS), they only account for whether death occurred, but not for the timing of death—which represents a limitation when assessing the long-term impact of an intervention.

The WR is an alternative outcome measure which addresses these limitations by integrating both efficacy and safety outcomes into a single hierarchical composite, ordered by clinical importance.^20^ The underlying pairwise comparison technique is conceptually independent of its application to multi-component composites: Howard et al.^21^ proposed essentially the same approach for the analysis of the ordinal mRS in acute-stroke RCTs over a decade ago, and Churilov et al.^22^ developed a generalised odds-ratio method in the same context. The extension of this technique to hierarchical composites combining outcomes of different types (time-to-event, ordinal, continuous) has been increasingly used in the past years.

Since its introduction in 2012, the WR has been used in 61 trials in the field of cardiology,^17^ and has recently emerged in stroke prevention trials, including carotid revascularisation studies.^23^ Regulatory agencies such as the US Food and Drug Administration have endorsed WR-based analyses and approved new pharmaceuticals and medical devices based on the results of such studies.^24^^,^^25^ An increasing number of studies prespecified the use of the WR in their primary analysis, as is the case for the interim analysis of the recently published ECST-2 (Second European Carotid Surgery Trial) trial.^23^ Recently, Yogendrakumar et al.^26^ used WR with hierarchical composite outcomes in their randomised trial of telemedicine models of care on a mobile stroke unit. However, the WR has not been applied in trials on EVT.

In this study, we applied the WR method to the TENSION trial^1^ data to investigate how different ways of specifying the hierarchy of the outcomes affect the trial conclusions. We found an overall WR of 1.61, indicating that, when patients treated with EVT + BMT and BMT alone are compared pairwise along the prespecified hierarchy, wins for the EVT + BMT patient occurred 1.61 times as often as wins for the BMT-alone patient. This effect size is directionally in line with the primary results of the TENSION trial and several prior randomised controlled trials which demonstrated the safety and efficacy of EVT in patients with LVO and large infarcts.^2^^,^^3^^,^^27^ To examine the contribution of each component to the overall WR and take account for tied pairs, a detailed analysis of the win difference is necessary. The win difference was 22.8%, with time to death contributing the most to the overall treatment effect (win difference: 15.7%). The subgroup analyses in our study revealed consistent treatment benefits across several subgroups, including both sexes, patients aged ≤ 80 years, patients with lower baseline disability (mRS 0–1), those conscious at presentation, those with ASPECTS 5, patients with M1 occlusions, and those not treated with intravenous thrombolysis. Our sensitivity analysis with an alternative hierarchy produced nearly identical results, which underscores the robustness of our findings.

Notably, there was a relatively high event rate in the TENSION trial (mortality at 12 months for EVT + BMT: 45%, BMT alone: 56%). In the context of WR analyses, this is particularly relevant as the number of events—and thus the number of ties—is directly correlated with the WR: With rare outcomes, there may be a large number of tied comparisons, eg, 94.7% in the REPRIEVE trial.^28^ As the WR does not account for ties, it may overestimate treatment effects in such settings, meaning that a large WR results from the treatment benefit in only a small proportion of the study population.^29^ It is therefore generally recommended to report the win difference, which accounts for ties and equals the “net treatment benefit.”^30^ While cardiology trials frequently suffer from a low event rate,^8^ acute ischaemic stroke due to LVO constitutes a disabling disease with a high number of events—making the WR particularly well suited for thrombectomy trials.

The high proportion of patients with death or dependency at follow-up in the TENSION trial (EVT + BMT: 67%, BMT alone: 77%) implies that secondary outcomes, such as quality-of-life measures were only available for a small group of patients, resulting in substantial missing data (133/253, 52.6%). However, these missing values constitute informative censoring, as they are rarely missing at random but rather because patients either died or suffered from severe disability. Hierarchical composite outcomes, recognising prior death or severe disability as worse outcome, appropriately account for these missing values, representing a major strength of the WR approach.

The hierarchical structure of WRs may also increase statistical efficiency: Because the WR constructs one overall inferential statement per subgroup analysis, it may reduce the problem of multiple testing. As the total number of observed events contributes to the power of an analysis, composite hierarchical outcome measures may increase the number of events and preserve statistical power in smaller sample sizes.^31^ The ECST-2 trial illustrates this advantage: originally powered for 2000 patients over 5 years, the investigators published an adequately powered interim analysis with only 253 patients using the WR.^23^ This illustrates how prespecifying the use of the WR method alongside conventional statistics can accelerate the dissemination of scientific results, terminate trials earlier and minimise participants’ exposure to inferior interventions once a clear therapeutic advantage has been demonstrated.

In the context of the TENSION trial under the primary hierarchy, a hypothetical RCT powered to detect a WR of 1.61 at a 2-sided α = 0.05 with 80% power would require a total of 193 patients under assumptions matching those observed here (win probability ≈ 0.604, allocation ratio *k* = 125/253 = 0.494 and tie proportion ≈ 0.02) and under simplified conditions that do not account for loss to follow-up, censoring or covariate adjustment.^32^ This sample size is much lower than the planned sample size of 665 patients in the TENSION study protocol, and broadly comparable to those of recent thrombectomy RCTs. Given the high event rate and low number of ties, this suggests that WR-based trial designs are operationally feasible in acute large-infarct stroke. However, any formal power analysis would require a prospectively co-designed hierarchy rather than one derived post-hoc from these data.

While the hierarchical structure of WRs has several advantages, it depends highly on the choice and ordering of outcomes. In cardiology trials, the hierarchical ordering of composite outcomes has frequently been a matter of debate: What is more severe—to suffer from acute limb ischaemia, myocardial infarction or ischaemic stroke? In most studies—as well as in ours—the hierarchy of outcome was predefined by the investigators. However, the hierarchy of perceived clinical event severity remains inherently subjective. While most clinicians would agree on prioritising survival over functional status, some patients may prefer quality of life over survival, thus potentially changing universally accepted ranking schemes. Future studies may integrate patient input to mitigate this issue and reflect patients’ priorities in functional outcomes. Unlike cardiology trials where hierarchical outcomes often occur independently, stroke trial outcomes are frequently correlated, which may complicate the interpretation of WR-based analyses. Our sensitivity analysis prioritising time to death over mRS revealed that most of the treatment effect in the TENSION trial was attributable to reduced mortality rather than improved functional outcome. Positioning mRS as the highest-ranked outcome obscured this distinction, as death is subsumed within the mRS as a single category: the win difference of 23.2% for functional outcome in the sensitivity analysis corresponds approximately to the sum of win differences observed in the primary analysis (15.7% for time to death and 6.6% for mRS). This finding demonstrates how re-ordering a hierarchical outcome may help gain information by defining the contribution of components to an integrative measure. Concerns regarding competing and correlated outcomes are not unique to WR-based analyses but rather constitute a general limitation of clinical trials, namely that—at least with conventional statistical methods—we cannot observe counterfactuals. While we cannot directly observe SAEs in deceased patients, SAEs are causally linked to higher-ranking outcomes as they may shorten survival or worsen functional outcomes. Consequently, the impact of SAEs may be partially captured within the mortality and mRS components. Similarly, EQ-5D assessment is restricted to survivors and may be confounded by preceding SAEs. In addition, as survival appears to be the dominant driver of the overall result, the extent to which quality-of-life differences captured by EQ-5D meaningfully contribute to the treatment effect is likely limited and survival may disproportionately influence the overall signal.

The choice of outcome measure is critical for the success of a trial. In the recently published ESCAPE-MeVO^33^ and DISTAL^34^ trials, a different specification of the primary endpoint may have significantly influenced the results. Patients with medium- and distal-vessel occlusion stroke frequently present with only minor deficits and tend to achieve favourable outcomes with BMT. In these cases, the benefits of thrombectomy may be more subtle, such as improved speech or fine motor skills; however, established outcome measures such as the mRS suffer from a lack of granularity to reflect modest but clinically relevant improvements.^6^ In studies with neutral findings, a reanalysis of trial data using the WR method may provide a more differentiated view on patient outcomes.

The mRS is—and will remain—a fundamental primary outcome in stroke trials, owing to its simplicity, intuitive interpretability for patients, long-standing validation and broad regulatory acceptance. Ordinal mRS analysis directly characterises the distribution of functional outcomes at a fixed time point and provides effect estimates interpretable on the scale of the outcome itself. In the present study, we demonstrate that hierarchical composite outcomes may offer advantages for the interpretation of trial results and may complement ordinal mRS analysis in various aspects: the WR incorporates the timing of death, which is discarded when mortality is encoded as mRS 6; it handles informatively missing quality-of-life data coherently, since pairs resolve at higher levels of the hierarchy before EQ-5D is evaluated; it integrates outcomes of different types (time-to-event, ordinal, continuous) that cannot be combined on the single ordinal scale of the mRS and it weights components by prespecified clinical priority. These properties come at the cost of a summary statistic that may be less directly interpretable at the patient level than the common or generalised odds ratio. Our findings therefore suggest that the WR may be reported alongside, rather than instead of, conventional ordinal mRS analyses, and support the prespecification of complementary WR-based analyses alongside conventional outcome analyses in future neurointerventional trials.

### Strengths and limitations

Our analysis has several strengths, including that it is derived from a multicentre randomised controlled trial. Methodologically, we evaluated the implications of different permutations of the components of a hierarchical composite outcome influence the interpretation of trial results.

However, there are several limitations. First, while the WR allows hierarchical prioritisation of clinically important outcomes, its interpretation is dependent on the predefined hierarchy, and the selection of outcomes may lead to varying results. The hierarchy used in this study treats longer survival as preferable over functional state. In large-infarct stroke, where many survivors remain at mRS 5, some patients and families may not regard prolonged survival with severe dependency as a desirable outcome. To mitigate this issue, we conducted sensitivity analyses with alternative hierarchical outcomes. However, while a sensitivity analysis placing EQ-5D at the top of the hierarchy would have been of interest in this regard, it may not be informative in the present dataset, because EQ-5D is undefined in deceased patients (52.6% missing at 12 months) and the majority of pairwise comparisons would therefore resolve on a lower-ranked component. We acknowledge that the hierarchy used here was investigator-specified for the purposes of this study. Construct validity of hierarchical composite outcomes ultimately requires prospective co-design with people with lived experience of stroke, their families and clinicians, before the results are known; and no post-hoc analysis of a completed trial, however transparently conducted, can substitute for that process. Our findings are intended to demonstrate how hierarchy choice affects WR-based inference in acute-stroke data and serve as empirical motivation for prospective co-design in future neurointerventional trials. Second, although a WR integrates multiple endpoints, it remains a summary statistic and does not replace the need for detailed reporting of individual outcome components. In addition, it does not directly translate into conventional measures of treatment effect, may obscure trade-offs between outcomes—particularly where mortality and functional status diverge—and does not inherently adjust for baseline differences. Third, while the WR provides a structured hierarchical assessment of net treatment benefit at the population level, it does not estimate individual treatment effects. Individualised benefit-harm estimation would be necessary to support more nuanced treatment decisions. Finally, although increasingly used in cardiology clinical trials, the WR remains a relatively new statistical approach, and clinical interpretation may be less familiar compared with classical time-to-first event analysis. As the WR does not translate directly into a patient-level probability of benefit, it may rather serve as an additional measure for the clinician to interpret the trial results. Broader methodological dissemination and prospective validation in future trials will be essential to establish its role in the analysis of stroke outcomes.

## Conclusion

The present study illustrates the value of the WR as an informative measure to evaluate outcomes after EVT in acute ischaemic stroke with large infarct. Including additional outcomes beyond the original study’s primary endpoint, our results revealed that the treatment effect was substantially derived from a benefit in survival. Our findings further encourage the complementary use of hierarchical composite outcomes in future neurointerventional trials.

## Supplementary Material

Supplement_aakag063

## Data Availability

Data are available from the corresponding author upon reasonable request. All code for data pre-processing, modelling and figure generation is available from the corresponding author upon reasonable request.

## ClinicalTrials.gov identifier

NCT03094715.
