# Supplementary material for: Hierarchical composite outcomes in acute ischaemic stroke with large infarct: a win ratio analysis of the TENSION trial
Source: Eur Stroke J. 2026 Jun 18;11(6):aakag063. doi: 10.1093/esj/aakag063 (PMC13278760; doi:10.1093/esj/aakag063)
Supplement: Supplement_aakag063 [file supplement_aakag063.docx]

**Supplement**

**Supplementary Figure S1:** Hierarchical structure of the WR Analysis with an alternate hierarchical composite outcome (1 = mRS at 12 months, 2 = time to death, 3 = any serious adverse event, 4 = EQ-5D at 12 months)


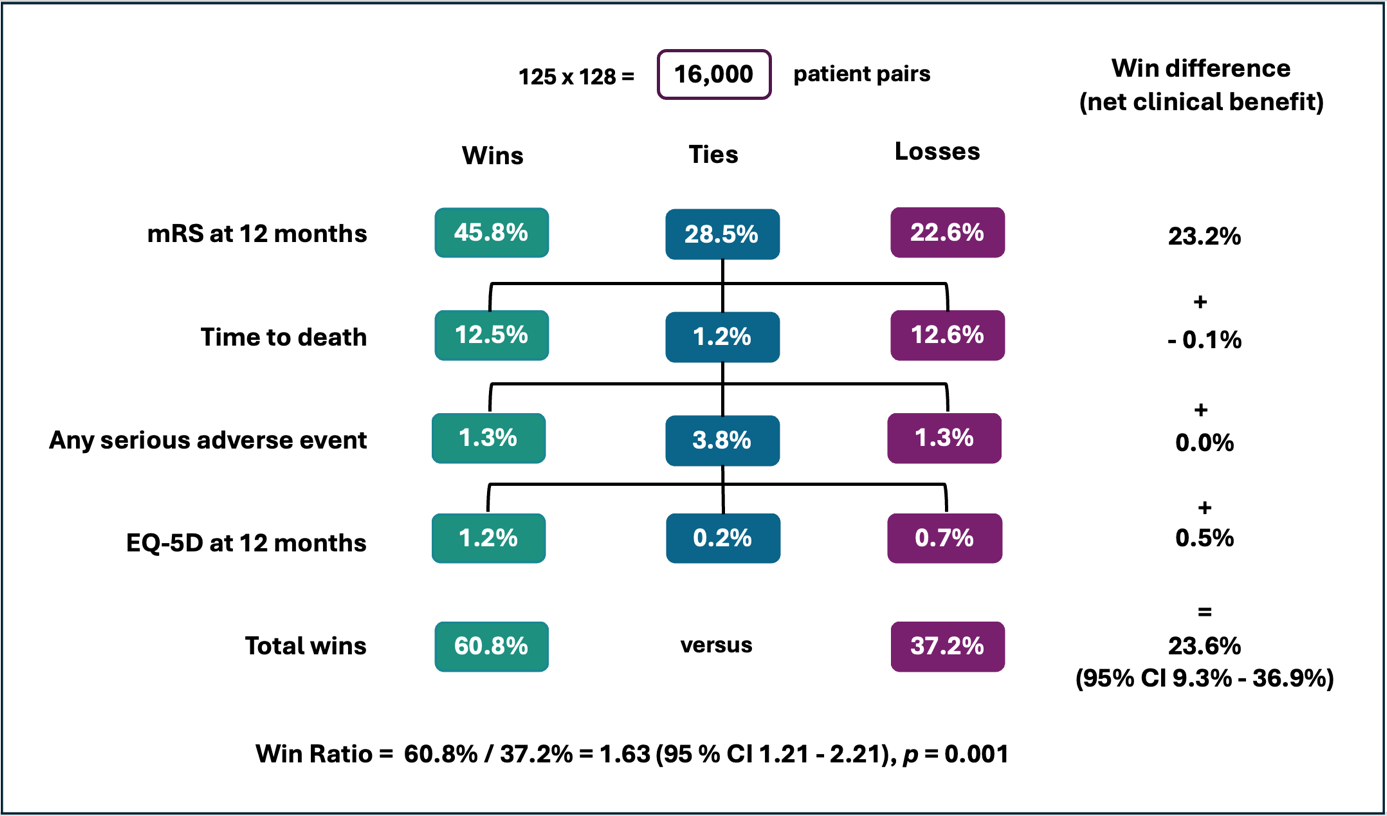


**Supplementary Figure S2:** Hierarchical structure of the WR Analysis with an alternate hierarchical composite outcome (1 = mRS at 12 months, 2 = time to death, 3 = EQ-5D at 12 months, 4 = any serious adverse event)


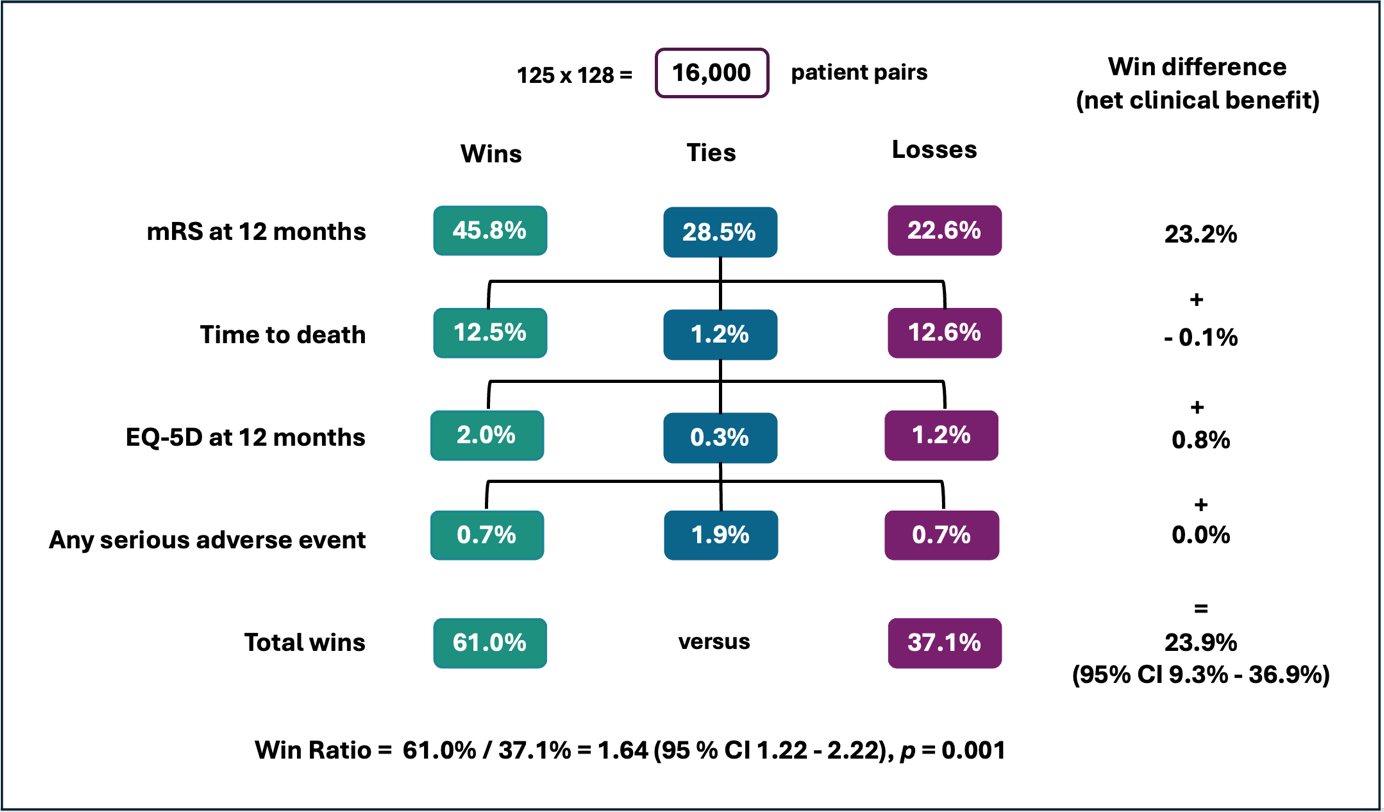


**Supplementary Table S1:** Unstratified and stratified WR Analysis for EVT+BMT versus BMT alone with alternate hierarchical composite outcome (1 = mRS at 12 months, 2 = time to death, 3 = any serious adverse event (SAE), 4 = EQ-5D at 12 months)

| **Subgroup** | **WR** | **95 % CI lower** | **95 % CI upper** | **p-value** |
| --- | --- | --- | --- | --- |
| **Overall** | 1.63 | 1.21 | 2.21 | **0.001** |
| **Sex** |  |  |  |  |
| Male | 1.63 | 1.08 | 2.48 | **0.021** |
| Female | 1.65 | 1.06 | 2.56 | **0.025** |
| **Age** |  |  |  |  |
| ≤ 70 | 1.84 | 1.15 | 2.95 | **0.012** |
| 71–80 | 1.98 | 1.15 | 3.41 | **0.014** |
| > 80 | 1.03 | 0.57 | 1.87 | 0.926 |
| **Baseline mRS** |  |  |  |  |
| 0-1 | 1.68 | 1.21 | 2.34 | **0.002** |
| ≥ 2 | 1.84 | 0.86 | 3.94 | 0.115 |
| **Consciousness at arrival** |  |  |  |  |
| Fully awake | 2.03 | 1.38 | 2.98 | **< 0.001** |
| Somnolent/coma | 1.21 | 0.75 | 1.97 | 0.434 |
| **ASPECTS value** |  |  |  |  |
| 3 | 1.29 | 0.78 | 2.16 | 0.322 |
| 4 | 1.66 | 0.99 | 2.81 | 0.056 |
| 5 | 2.05 | 1.20 | 3.52 | **0.009** |
| **Occlusion site** |  |  |  |  |
| ICA (t/i/e) | 1.20 | 0.72 | 2.0 | 0.485 |
| M1 | 1.87 | 1.33 | 2.63 | **< 0.001** |
| **Thrombolysis** |  |  |  |  |
| Thrombolysis | 1.21 | 0.75 | 1.96 | 0.441 |
| No Thrombolysis | 2.06 | 1.39 | 3.04 | **< 0.001** |

ASPECTS, Alberta Stroke Program Early CT Score; CI, confidence interval; EVT, endovascular thrombectomy; ICA, internal carotid artery; M1, M1 segment of the middle cerebral artery; t/i/e, terminal/intracranial/extracranial; WR, Win Ratio.

**Supplementary Table S2:** Unstratified and stratified Win Ratio Analysis for EVT+BMT versus BMT alone with alternate hierarchical composite outcome (1 = mRS at 12 months, 2 = time to death, 3 = EQ-5D at 12 months, 4 = any serious adverse event (SAE))

| **Subgroup** | **WR** | **95 % CI, lower** | **95 % CI, upper** | **p-value** |
| --- | --- | --- | --- | --- |
| **Overall** | 1.65 | 1.22 | 2.22 | **0.001** |
| **Sex** |  |  |  |  |
| Male | 1.64 | 1.08 | 2.49 | **0.019** |
| Female | 1.67 | 1.07 | 2.58 | **0.023** |
| **Age** |  |  |  |  |
| ≤ 70 | 1.87 | 1.16 | 2,99 | **0.010** |
| 71–80 | 2.00 | 1.16 | 3.45 | **0.013** |
| > 80 | 1.03 | 0.57 | 1.87 | 0.926 |
| **Baseline mRS** |  |  |  |  |
| 0-1 | 1.68 | 1.21 | 2.35 | **0.002** |
| ≥ 2 | 1.88 | 0.88 | 4.02 | 0.104 |
| **Consciousness at arrival** |  |  |  |  |
| Fully awake | 2.03 | 1.38 | 2.99 | **< 0.001** |
| Somnolent/coma | 1.23 | 0.76 | 2.00 | 0.405 |
| **ASPECTS value** |  |  |  |  |
| 3 | 1.31 | 0.78 | 2.18 | 0.304 |
| 4 | 1.70 | 1,01 | 2.87 | **0.047** |
| 5 | 2.01 | 1.18 | 3.43 | **0.011** |
| **Occlusion site** |  |  |  |  |
| ICA (t/i/e) | 1.20 | 0.72 | 1.99 | 0.492 |
| M1 | 1.88 | 1.34 | 2.65 | **< 0.001** |
| **Thrombolysis** |  |  |  |  |
| Thrombolysis | 1.21 | 0.75 | 1.97 | 0.433 |
| No Thrombolysis | 2.06 | 1.39 | 3.05 | **< 0.001** |

ASPECTS, Alberta Stroke Program Early CT Score; CI, confidence interval; EVT, endovascular thrombectomy; ICA, internal carotid artery; M1, M1 segment of the middle cerebral artery; t/i/e, terminal/intracranial/extracranial; WR, Win Ratio.
